# More Evidence for PBDEs as Neurotoxicants: Cohort Study Corroborates Earlier Findings

**DOI:** 10.1289/ehp.122-A221

**Published:** 2014-08-01

**Authors:** Kellyn S. Betts

**Affiliations:** Kellyn S. Betts writes about environmental contaminants, hazards, and technology for solving environmental problems for publications including *EHP* and *Environmental Science & Technology*.

Children from the Midwest involved in a prospective study are the third U.S. birth cohort to show strikingly consistent associations between prenatal exposure to polybrominated diphenyl ether (PBDE) flame retardants and impaired performance on neurodevelopment tests later in childhood.^1^ As in the earlier studies involving birth cohorts from New York City^2^ and California,^3^ the children’s mothers had PBDE levels consistent with average U.S. adult exposure. The concordance of the three studies strongly supports the likelihood that PBDEs are developmental neurotoxicants, according to the authors of the new work.

PBDEs were widely used between the 1970s and 2004 in U.S. consumer goods including furniture, carpet padding, and electronic devices. The main PBDEs found in humans are from the now-banned penta and octa mixtures used in polyurethane foam padding and electronics enclosures, respectively.^4^ People can accumulate PBDEs in their bodies through food and inadvertent ingestion, inhalation, and dermal absorption of house dust.^5^^,^^6^

**Figure:**
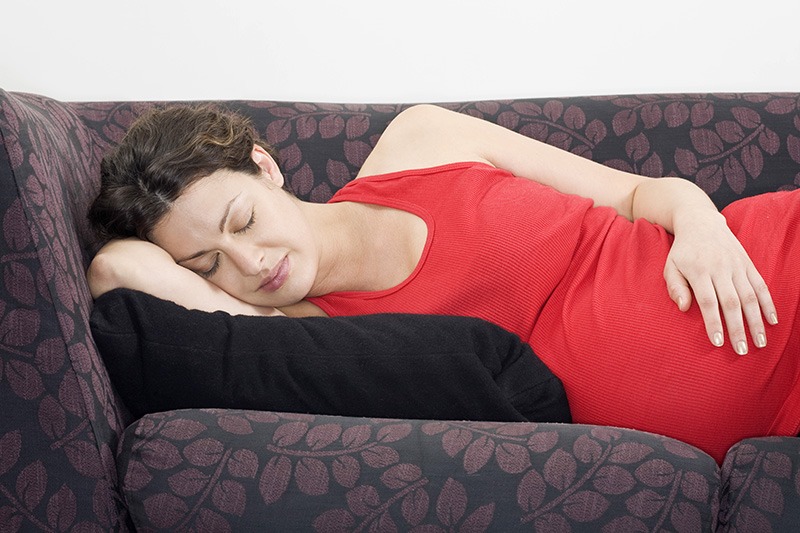
BDE-47 is a major component of pentaBDE, a flame retardant mixture still found in older foam cushioning. © bikeriderlondon/Shutterstock

The new research involved participants in the Health Outcomes and Measures of the Environment (HOME) Study, a prospective birth cohort of women living around Cincinnati, Ohio, who were pregnant between 2003 and 2006. All 309 mothers provided blood samples at about 16 weeks’ gestation, and all had detectable levels of BDE-47, one of the most commonly detected PBDE congeners and the focus of the new study’s primary analysis. Their children underwent yearly developmental and/or behavioral assessments through age 5, plus IQ testing at the last followup.

After adjusting for potential confounders, including maternal IQ, the researchers found a statistically significant inverse relationship between mothers’ BDE-47 concentrations and their children’s IQ scores, says coauthor Aimin Chen, an epidemiologist at the University of Cincinnati College of Medicine. In repeated measurements of cognition, a 10-fold increase in maternal BDE-47 concentration was associated with a 4.5-point reduction in IQ score,^1^ consistent with decrements reported for the New York City^2^ and California^3^ cohorts. Exposure was also associated with a 3.3-point increase in hyperactivity score.^1^

“What’s really important is the consistency in the results across three very different populations, [which is] pretty amazing,” says Linda Birnbaum, director of the National Institute of Environmental Health Sciences. She observes that the new study’s use of maternal serum taken during the first part of the second trimester—earlier than in the other cohorts—suggests outcomes could be linked to earlier *in utero* exposures.

Small shifts in a population’s IQ matter, says coauthor Bruce Lanphear of Simon Frasier University in Vancouver, Canada. Lanphear, who is the senior principal investigator of the HOME Study, explains that a 5-point shift downward in the mean IQ of U.S. children would result in an additional 3.4 million children who are considered intellectually disabled or mentally retarded,^7^ with corresponding costs in treatment, special education, and lost lifetime earnings. “There isn’t a published estimate of the cost of PBDE toxicity,” he says, “but based on what we know about the impact and costs of lead toxicity, I would expect the cost of PBDEs to exceed $10 billion annually in the United States.”

The prospective cohort design with repeated follow-up visits is a major strength of the new study. Its weaknesses include the lack of PBDE measurements in the children’s serum, which other research shows can be higher than maternal levels.^3^^,^^8^ Julie Herbstman of Columbia University’s Mailman School of Public Health, lead author of the earlier New York City study,^2^ also points out that the half-life of BDE-47 in humans is thought to be on the order of years, so it’s hard to isolate the timing of exposure.

There is some evidence that PBDE levels are declining in the U.S. population.^9^ Even so, “pretty much everyone still has penta- and octa-containing products in their home,” says Herbstman. Typically, the types of products that contained these compounds—such as couches—are not replaced frequently.

Because PBDEs’ persistence allows them to remain in indoor environments for a long time, Chen recommends that prospective mothers and other concerned individuals wash their hands frequently, vacuum with HEPA filters, and use wet cloths to dust furniture. The nonprofit Environmental Working Group also recommends replacing any foam-containing item with a ripped cover or cushioning that is misshapen or breaking down.^10^
